# Protein turnover measurement using selected reaction monitoring-mass spectrometry (SRM-MS)

**DOI:** 10.1098/rsta.2015.0362

**Published:** 2016-10-28

**Authors:** Stephen W. Holman, Dean E. Hammond, Deborah M. Simpson, John Waters, Jane L. Hurst, Robert J. Beynon

**Affiliations:** 1 Centre for Proteome Research, Department of Biochemistry, Institute of Integrative Biology, University of Liverpool, Crown Street, Liverpool L69 7ZB, UK; 2 Cellular and Molecular Physiology, Institute of Translational Medicine, University of Liverpool, Crown Street, Liverpool L69 3BX, UK; 3 Mammalian Behaviour and Evolution Group, Department of Evolution, Ecology and Behaviour, Institute of Integrative Biology, University of Liverpool, Leahurst Campus, Neston CH64 7TE, UK

**Keywords:** protein turnover, proteomics, mass spectrometry, quantification, SRM, MS1

## Abstract

Protein turnover represents an important mechanism in the functioning of cells, with deregulated synthesis and degradation of proteins implicated in many diseased states. Therefore, proteomics strategies to measure turnover rates with high confidence are of vital importance to understanding many biological processes. In this study, the more widely used approach of non-targeted precursor ion signal intensity (MS1) quantification is compared with selected reaction monitoring (SRM), a data acquisition strategy that records data for specific peptides, to determine if improved quantitative data would be obtained using a targeted quantification approach. Using mouse liver as a model system, turnover measurement of four tricarboxylic acid cycle proteins was performed using both MS1 and SRM quantification strategies. SRM outperformed MS1 in terms of sensitivity and selectivity of measurement, allowing more confident determination of protein turnover rates. SRM data are acquired using cheaper and more widely available tandem quadrupole mass spectrometers, making the approach accessible to a larger number of researchers than MS1 quantification, which is best performed on high mass resolution instruments. SRM acquisition is ideally suited to focused studies where the turnover of tens of proteins is measured, making it applicable in determining the dynamics of proteins complexes and complete metabolic pathways.

This article is part of the themed issue ‘Quantitative mass spectrometry’.

## 1. Introduction

Protein abundance (either in relative or absolute terms) is now routinely measured in proteomics [1]. For most intracellular proteins, the abundance is the balance of two dynamic metabolic processes, protein synthesis and protein degradation, which collectively constitute the process of ‘protein turnover’ [2]. Any change in protein abundance, whether physiological or pathological, must reflect a shift in the balance between these two processes, and thus, while it is valuable to understand differences in protein level, it is also important to understand the changes in protein turnover whereby the protein pool is influenced. For example, a protein could decrease in abundance through reduced synthesis, through enhanced degradation, or by a simultaneous adjustment of both processes. It is evident that a full understanding of the response requires a clear understanding of the relative contribution of the two processes. Moreover, even a protein in an unchanging steady-state concentration is undergoing continual replacement (turnover) and it could be argued that a full understanding of a steady-state proteome would include accurate data on the rate at which that protein pool is being replaced inside the cell.

Historically, protein turnover has been measured using radioisotopically labelled amino acids or simple metabolic precursors such as ^14^C-bicarbonate [2]. However, the low extent of radiolabelling that could be attained meant that most studies were focused on the turnover of the total tissue protein pool rather than of individual proteins: it was only possible to measure specific turnover rates for a few highly abundant proteins [3,4]. More recently, the analytical capabilities of proteomics, employing liquid chromatography-mass spectrometry (LC-MS), have introduced the opportunity to measure turnover at the individual protein level using proteolytic peptides as surrogates, as well as avoiding the use of radioisotopes.

Whether the system is in steady-state, or changing between different states, it is axiomatic that turnover can only be measured by monitoring the flux of a tracer through the protein pool. To acquire these data for multiple proteins, the only feasible approach is to use stable isotope precursors as the monitored label. The mass resolving property of MS means that the introduction or removal of stable isotope label is distinguishable, and thus measurable, in *m*/*z* space. Isotopic enrichment of the protein pool can be achieved using heavy isotope labelled amino acids such as leucine [5], valine [6], lysine [7,8] or arginine [8], or metabolic precursors such as [^15^N]-ammonium sulfate [9] or [^2^H]_2_O [10].

The majority of proteomic studies of protein turnover have focused on non-targeted (shotgun) measurement on a large scale [11]. Investigations have been conducted in both prokaryotic [12,13] and eukaryotic cells in culture [7,8] using dynamic [5] and pulsed [14] SILAC approaches. Several studies have described sub-cellular measurements of protein turnover targeting specific organelles [15,16] to provide spatial resolution of synthesis and degradation [17], and recently, methods to measure protein turnover in body fluids have been described [18,19]. In addition, proteomics has been applied to measure protein turnover in tissues from whole animals [20,21]. All of these approaches have emphasized global acquisition of turnover rates using data-dependent acquisition (DDA). This mode of acquisition is straightforward to implement, requires little method development time and allows quantitative measurements of thousands of proteins in single analyses [22]. DDA involves selecting precursor ions for tandem mass spectrometry (MS/MS), from which sequence can be determined, based on their signal intensities; modern instrumentation is capable of acquiring high-quality high mass resolution product ion spectra from between the top 12 to 20 most intense peptide ions detected in a given full scan mass spectrum [22–26]. The precursor ion selection based on signal intensity means that DDA analyses tend to be biased towards abundant peptides, and thus proteins. Low abundance proteins are therefore often underrepresented in DDA analyses, and targeted proteomics approaches, such as selected reaction monitoring (SRM) [27], are frequently required to quantify these trace analytes [28]. The SRM approach involves measuring product ions that are diagnostic of specific peptides from proteins of interest, preselected by the analyst. This focused strategy typically yields enhanced sensitivity (by orders of magnitude) and selectivity, leading to higher quality data. Careful experimental design is required for a SRM experiment, which can be time-consuming. However, repositories of optimal product ions to monitor for specific peptides expedite this stage significantly [29]. The benefits obtained by SRM in quantitative studies are equally realizable in protein turnover measurements, but to date, there has been little consideration given to the benefits in terms of data quality that can be leveraged through the use of targeted MS methods for data acquisition in protein turnover studies. Indeed, the application of targeted MS has lagged behind DDA methods in breadth of usage for measuring protein turnover, although a few studies have emerged recently [30–34]. In this study, the measurement of protein turnover using SRM is explored and compared to data acquired using ‘traditional’ intact peptide precursor ion signal intensity (MS1) with DDA shotgun analysis. We show that SRM quantification provides better sensitivity and selectivity compared with MS1 analysis, allowing improved and more confident measurement of protein turnover.

## 2. Experimental

### (a) Protein and peptide selection

Tryptic peptides for monitoring in SRM assays were selected from four tricarboxylic acid (TCA) cycle proteins of high abundance (between top 5 and 25% of the mouse liver proteome from the integrated dataset in PaxDb) [35]. Non-unique peptides in the reviewed UniProt canonical and isoform database of *Mus musculus* (accessed 25 November 2015) were removed using Skyline [36]. All arginine-terminating and non-lysine-containing protein C-terminal peptides were excluded from consideration due to the absence of an isotopic label in these proteolytic fragments, and no missed cleavage peptides were considered. N-terminal peptides were also excluded due to potential for exoproteolytic fraying [37].

### (b) Experimental animals

Eleven adult male C57BL/6JOlaHsd mice (Harlan UK Ltd, Shardlow, UK) aged 14 months at the start of the experiment were housed individually in 48 × 15 × 15 cm polypropylene cages (NKP Cages Ltd, Coalville, UK). Each cage contained substrate (Corn Cob Absorb 10–14 substrate), paper wool nest material and environmental enrichment. Food (LabDiet 5002 Certified Rodent Diet, Purina Mills, St Louis, USA) and water were provided ad libitum. The mice were maintained on a reversed photo-period (12 L : 12 D; lights on at 20:00) and at 20–22°C. Standard laboratory diet was replaced with a semi-synthetic diet with the inclusion of [^13^C_6_]lysine at a relative isotope abundance (RIA) of 0.5. The dietary pellets were dissociated with water containing the dissolved [^13^C_6_]lysine to form a thick paste and mixed extensively. Once homogeneous, the paste was then extruded into strips 1 cm across and dried in a commercial foodstuff drying oven at 40°C. The mice had access to the labelled diet for varying amounts of time: 0, 1, 2, 3, 4, 6, 9, 12, 17, 22 or 30 days exposure to the diet. The day that the animals were introduced to the labelled diet was staggered in order for all culls and dissections to take place on the same day. All mice were humanely killed on day 30 and dissected to recover the liver tissue from each animal. All resected tissue was frozen at −80°C prior to analysis.

### (c) Sample preparation

A small section of liver tissue was removed and further cut into small pieces prior to homogenization in 1 ml of lysis buffer (7 M urea, 2 M thiourea, 2% (w/v) CHAPS, 5 mM DTT) using a Precellys lysis kit (Stretton Scientific Ltd, Stretton, UK). Total protein extracted was quantified using a Bradford assay. Proteins (200 µg) were reduced, alkylated and digested with trypsin using a modified version of the filter-aided sample preparation (FASP) approach [38,39].

### (d) Liquid chromatography-mass spectrometry/mass spectrometry analysis

Non-targeted MS1-DDA analyses were conducted on a QExactive HF quadrupole-Orbitrap mass spectrometer [40,41] coupled to a Dionex Ultimate 3000 RSLC nano-liquid chromatograph (Hemel Hempstead, UK). Peptides (1 µg) from each time-point were loaded in technical triplicates onto a trapping column (Acclaim PepMap 100 C18, 75 µm × 2 cm, 3 µm packing material, 100 Å) using a loading buffer of 0.1% (v/v) trifluoroacetic acid, 2% (v/v) acetonitrile in water for 7 min at a flow rate of 12 µl min^−1^. The trapping column was then set in-line with an analytical column (EASY-Spray PepMap RSLC C18, 75 µm × 50 cm, 2 µm packing material, 100 Å) and the peptides eluted using a linear gradient of 96.2% A (0.1% (v/v) formic acid) : 3.8% B (0.1% (v/v) formic acid in water : acetonitrile (80 : 20) (v/v)) to 50% A : 50% B over 90 min at a flow rate of 300 nl min^−1^, followed by washing at 1% A:99% B for 8 min and re-equilibration of the column to starting conditions. The column was maintained at 40°C, and the effluent introduced directly into the integrated nano-electrospray ionization source operating in positive ion mode. The mass spectrometer was operated in DDA mode with survey scans between *m*/*z* 350 and 2000 acquired at a mass resolution of 60 000 (full width at half maximum) at *m*/*z* 200. The maximum injection time was 100 ms, and the automatic gain control was set to 3 × 10^−6^. The 16 most intense precursor ions with charge states of between 2+ and 5+ were selected for MS/MS with an isolation window of 1.2 *m/z* units. The maximum injection time was 45 ms, and the automatic gain control was set to 1 × 10^−5^. Fragmentation of the peptides was by higher-energy collisional dissociation using a stepped normalized collision energy of 28–30%. Dynamic exclusion of *m*/*z* values to prevent repeated fragmentation of the same peptide was used with an exclusion time of 20 s. Raw data files were imported into Progenesis QI for Proteomics v. 2.0 (Waters Ltd, Newcastle-upon-Tyne, UK) for peak detection and alignment. Data from all time-points were combined and searched against the UniProt canonical and isoform database of *Mus musculus* (accessed 25 November 2015) using Mascot v. 2.4.1 (Matrix Science, London, UK). The precursor ion mass tolerance was set to 10 ppm, and the product ion tolerance to 10 mmu. Carbamidomethylation was selected as a fixed modification, and oxidized methionine and [^13^C_6_]lysine were set as variable modifications. Two missed cleavages were permitted. The false discovery rate at the peptide level was set to 1%, and peptide identifications were further filtered using a peptide ion score of 23 or greater (score indicating identity or extensive homology). Peptides for which both the light and heavy isotopologue were identified were included in the downstream analysis. Raw data were imported into Skyline, the monoisotopic peak of each precursor ion extracted using the software's defined settings for MS1 quantification, peak picking manually checked and correlated in terms of retention time with the identifications from Mascot, peaks smoothed using Savitsky–Golay smoothing, and integrated peak areas exported for further processing.

SRM experiments were conducted on a Waters TQ-S tandem quadrupole mass spectrometer coupled to a Waters nanoACQUITY nano-liquid chromatograph (Elstree, UK). LC and MS conditions were as previously described for SRM analysis [28,42,43]. Briefly, peptides (1 µg) from each time-point were loaded in technical triplicates onto a trapping column (Symmetry C18, 180 µm × 2 cm, 5 µm packing material) in 99.9% A (0.1% (v/v) formic acid):0.1% B (0.1% (v/v) formic acid in acetonitrile) for 3 min at a flow rate of 5 µl min^−1^. The trapping column was then set in-line with an analytical column (HSS T3 C18, 75 µm × 152 cm, 1.8 µm packing material) and the peptides eluted using a linear gradient of 97% A:3% B to 60% A:40% B over 60 min at a flow rate of 300 nl min^−1^, followed by washing at 95% B for 5 min and re-equilibration of the column to starting conditions. The column was maintained at 35°C, and the effluent introduced into a nano-electrospray ionization source operating in positive ion mode. Both quadrupoles were operated at unit mass resolution. Transitions were selected using a combination of in-house acquired data from the non-targeted DDA analysis, and publicly available peptide spectral libraries from NIST (www.peptide.nist.gov) for all peptides passing the criteria outlined above. This led to a total of between 8 and 18 peptides being targeted for each protein. Between 4 and 13 product ions for each peptide were selected, and the samples analysed using an unscheduled SRM method to determine the two-to-four transitions with the highest signal-to-background ratios to monitor in the final quantitative assay. The peptides were separated into two categories based on their ionization efficiency: ‘good’ and ‘poor’. Methods were retention time scheduled [44] into 3 min windows to achieve dwell times per transition of ≥16 ms for good ionization efficiency peptides, and ≥28 ms for poor ionization efficiency peptides. Raw data were imported into Skyline, peak picking manually checked, peaks smoothed using Savitsky–Golay smoothing, and integrated peak areas exported for further processing.

### (e) Data processing

Peak areas were used to determine RIA values at each time-point using the equation

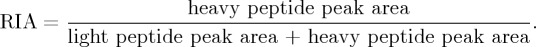

Average RIA values from the three replicate injections were calculated for each peptide at each time-point. These data were then processed using in-house developed R scripts as detailed previously to generate labelling curves [21].

## 3. Results and discussion

**Figure 1.**
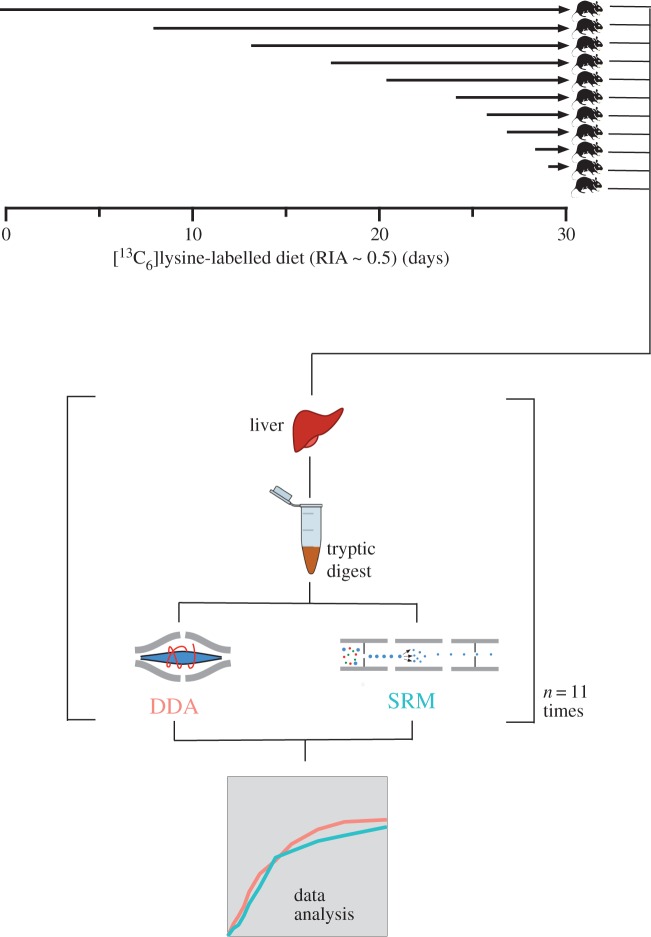
Overview of study workflow. Eleven mice had access to a semi-synthetic [^13^C_6_]lysine-containing diet (RIA = 0.5) for varying amounts of time (0, 1, 2, 3, 4, 6, 9, 12, 17, 22 or 30 days). All mice were humanely killed on day 30. The liver from each mouse was dissected. Liver tissue was homogenized by bead beating in the presence of lysis buffer. Proteins were reduced, alkylated and digested with trypsin using the FASP approach. Tryptic peptides were analysed by LC-MS using either quadrupole-Orbitrap (non-targeted) or tandem quadrupole (targeted) MS. (Online version in colour.)

This study sought to assess the quantitative performance of targeted SRM compared to the non-targeted MS1-based approach (with DDA for identification) typically used to measure protein turnover. A model system using C57BL/6JOlaHsd male adult mice was used (figure 1). Eleven mice were fed on a standard diet and transferred to a semi-synthetic diet containing [^13^C_6_]lysine at a RIA of 0.5 on different days. The entire cohort of mice was sacrificed on day 30, meaning that the duration of exposure to labelled amino acid varied between 0 and 30 days. Liver tissue from each animal was homogenized by bead beating in the presence of lysis buffer and processed using FASP to generate tryptic peptides. The samples were then analysed by LC-MS, using either MS1-DDA on a quadrupole-Orbitrap mass spectrometer or SRM on a tandem quadrupole mass spectrometer.

**Figure 2.**
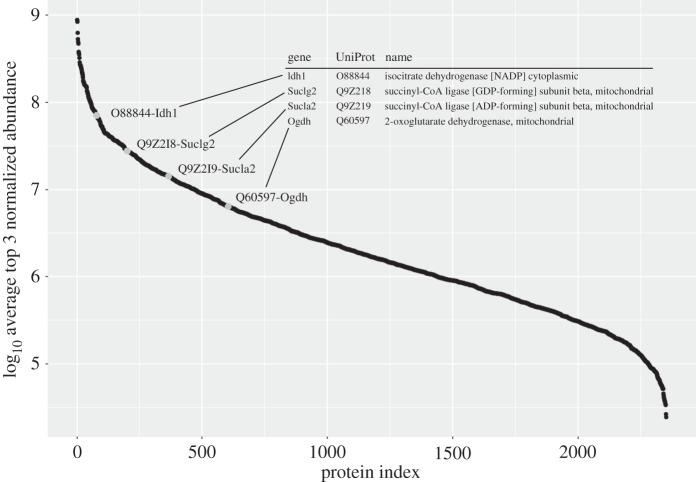
Label-free abundances of proteins used in this study. Label-free quantification of all proteins in the proteomics analysis was derived by calculation of the ‘top three’ normalized abundance, obtained from the average summed intensity of the three most intense peptides (calculated from triplicate analyses of day 0 sample using MS1-DDA analysis). The abundance values are plotted logarithmically (log_10_) as a function of protein index, ranked from the most to the least abundant. The proteins analysed for the MS1 and SRM analyses are highlighted in grey.

For the targeted analysis, four relatively abundant proteins from the TCA cycle were selected as exemplars to assess whether targeted quantification using SRM provided enhanced quantitative performance compared with the MS1 approach. The four proteins (Idh1, Suclg2, Sucla2 and Ogdh; figure 2) were selected on the basis that they occupied the upper-to-middle abundance range of protein expression in mouse liver, ranking 79th, 201st, 366th and 604th out of 2351 proteins in terms of top three label-free abundance [45]. These proteins were selected from this region of the abundance profile because we wished to examine multiple peptides in both MS1-DDA and SRM mode; proteins near the bottom of the abundance profile would not have yielded a satisfactory number of peptides for this study. For the MS1-DDA analyses, peptides were selected on the basis of lysine-termination and uniqueness within the proteome; for SRM, peptides were selected on the same criteria, in addition to suitable transitions being available, either from our own DDA data or by reference to published repositories. The peptides, and transitions used to monitor them, are presented in electronic supplementary material, table S1. For the four proteins, we were able to use between 5 and 12 peptides for MS1-DDA analyses and a further six to eight peptides for SRM. Thus, for many peptides, turnover curves could be generated using both approaches.

**Figure 3.**
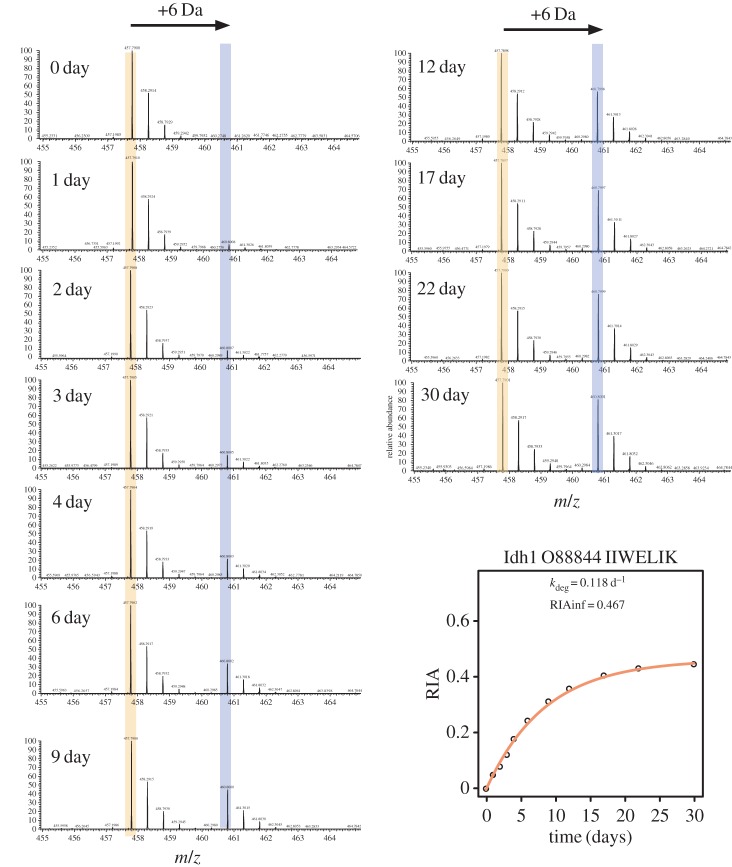
Generation of labelled isotopologues as a function of synthesis *de novo*. Mass spectra showing the appearance of the heavy isotopologue ([M + 2H]^2+^ ion, *m*/*z* 460.7992) of IIWELIK (from Idh1) over the course of the experiment, and the resulting labelling curve from the acquired data. The arrow denotes the additional 3.0101 *m/z* units introduced into the peptide due to the incorporation of [^13^C_6_]lysine (6.0201 Da) into newly synthesized protein molecules. From the intensity of the unlabelled (L) and labelled (H) peptides the relative isotope abundance (RIA) can be calculated as H/(H + L). (Online version in colour.)

As the ingested amino acid became incorporated into protein, the lysine-terminated peptides became progressively labelled. The RIA of the ingested amino acid was set experimentally to approximately 0.5, an approach that sustains palatability of the diet but which gives an acceptable dynamic range for labelling. Each lysine-terminated peptide was thus present in unlabelled and labelled forms, permitting the calculation of the RIA for that peptide at each time-point in the experiment. The time of labelling is a single exponential rise to plateau and the (RIA,t) data for each peptide can be analysed to yield the first order rate constant for that tissue (figure 3). Because the animals are adult and thus not growing, this is equivalent to the first order rate constant for degradation.

**Figure 4.**
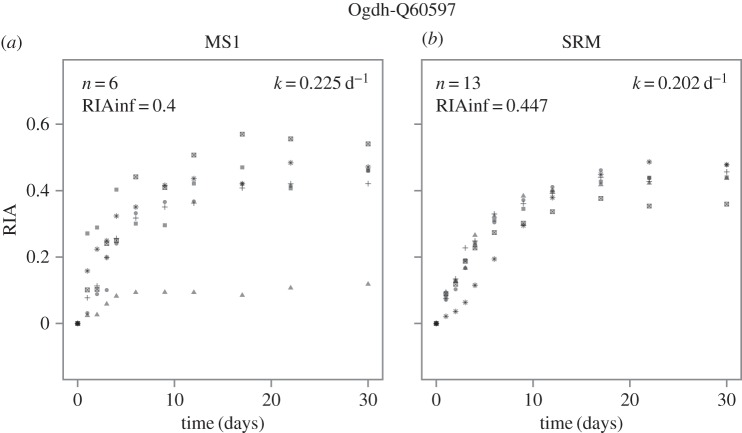
Labelling curves generated using quantitative MS data acquired by MS1 and SRM. For one protein (Ogdh) the labelling trajectory for (*a*) six (MS1) or (*b*) 13 (SRM) peptides was calculated on a per-peptide basis. Each datum is the average of RIA values determined for each peptide from triplicate analyses at each time-point.

As is evident from these spectra, there is potential for contribution to the ion intensity by contaminating peptides from other proteins that are isobaric in *m/z* space, and this can also be time-dependent, as the gradual shift from unlabelled to labelled peptides takes effect. This is evident by examination of the labelling profiles of all detectable lysine-terminated peptides from a single protein, for example Ogdh with six lysine-terminated peptides (figure 4*a*). Although several of the peptides yielded monotonic labelling profiles that were satisfactory, others generated profiles that were extremely noisy or which demonstrated systematic bias in the labelling outcome. For example, one peptide only attained an apparent labelling plateau of 0.15, which is impossible: all peptides from one protein should attain the same extent of labelling as the protein ‘sampled’ a uniformly labelled tRNA pool at the moment of synthesis, fleetingly short relative to the total labelling experiment. A more logical explanation is that a contaminant peptide was contributing to a signal for the light isotopologue that suppressed the contribution of the true unlabelled peptide.

By contrast, SRM quantification is capable of isolating a single peptide by a further tight selection on product ions derived from gas-phase dissociation of the precursor ion in a collision cell. Because most contaminant peptides would be unlikely to both contribute to the precursor ion current and also generate product ions equal in *m*/*z* values, this approach should yield enhanced data. Thus, SRM data for the same peptides (figure 4*b*) yielded labelling profiles that were much less noisy, and also trace a common labelling curve with a much enhanced similarity. In addition, the ability of SRM to generate clean isolation of specific peptides means that it was possible to recover turnover data from more peptides than by MS1-DDA. For the examples chosen here, more peptides were recovered than would strictly be necessary, but this allowed us to explore whether each peptide provided identical measures of the turnover rate constant.

For the four proteins analysed here, labelling profiles were obtained from multiple peptides either by precursor quantification (MS1) or by SRM (electronic supplementary material, figure S1). Even cursory inspection of the curves reveals the benefits for using targeted MS for measuring protein turnover. Firstly, all protein labelling profiles are described by more peptides using SRM compared with MS1 analysis (between six and eight additional peptides). This is because the duty cycle of the instrument is directed to acquisition of data for specific peptides, rather than operating in a ‘shotgun’ manner in an attempt to record data for as many peptides as possible [11]. The more efficient use of the instrument's duty cycle is manifested as an increased likelihood of detecting peptides of interest, which is particularly advantageous for those of low abundance or poor ionization efficiencies; the fewer ions generated by these analytes are more likely to be detected if the instrument is focused on recording data for these [27]. Electronic supplementary material, figure S1 also shows that SRM can give greater confidence in the measurement of protein degradation rate (*k*_deg_). The grey shading on each of the plots represents the 95% confidence intervals of the predicted nonlinear curve fit based on all of the quantified peptides. The confidence intervals demonstrate that the variance in the data acquired by SRM is either the same or lower than when MS1 data are used to measure turnover. This lower variance leads to a more accurate measurement of *k*_deg_, and greater confidence in the experimental determination of the rate of turnover. This is further emphasized when comparing *k*_deg_ measurement on a per-peptide basis, using the 30 peptides from four proteins that permitted quantification by both MS1 and SRM (electronic supplementary material, figure S2). Although some peptides yielded very similar *k*_deg_ values, irrespective of quantification method, for other peptides the discrepancy was more marked. The discrepant values were always associated with larger errors, consistent with curve fitting to data that were more variable. Of note, the errors of the parameter estimate are, in general, smaller for the SRM data compared with that acquired by MS1. This means that the determination of RIA at each time-point more closely fits the labelling curve when using SRM (labelling curves for all peptides quantified by both methods are shown in electronic supplementary material, figures S3 (MS1) and S4 (SRM)), giving enhanced confidence in both the data and the resultant recovery of turnover parameters.

A specific exemplar of the benefit of SRM-derived increased sensitivity is given in the electronic supplementary material, figure S5. By MS1 analysis, no strong evidence for the detection of the light : heavy pair of AAAQVLGNSGLFNK (from Sucla2) was observed in the retention time window in which the peptide was expected to elute (electronic supplementary material, figure S5*a*) (for comparison, an example of a peptide, SDYLNTFEFMDK, where both isotopologues were detected is shown in the electronic supplementary material, figure S6) [44]. Analysing the samples by SRM allows detection of the same peptide at each of the time-points, enabling description of a labelling curve that can be used to measure turnover of the parent protein (electronic supplementary material, figure S5*b*). The increased sensitivity of detection afforded by SRM therefore allows data to be recorded for more peptides. This increases certainty in the measurement of turnover rate, and allows peptides that produce inaccurate quantitative data to be identified and excluded from consideration. This study included mouse liver proteins of high abundance according to PaxDb, and supported by top three label-free quantification using the MS1 data acquired for the day 0 samples (no heavy label) (figure 2). These are in the range in which non-targeted MS1 quantification methods tend to perform well, but deeper proteome coverage can prove problematic using these strategies. The increased sensitivity of SRM will therefore allow measurement of turnover of low abundance proteins in complex proteomes that are inaccessible to MS1 quantification.

**Figure 5.**
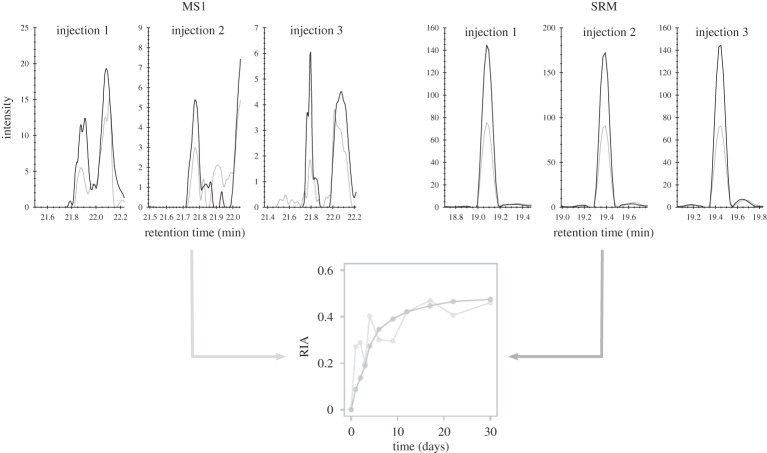
Comparative peptide chromatograms from MS1 and SRM. Triplicate MS1 reconstructed ion current chromatograms and triplicate SRM chromatograms for the light (black) and heavy (grey) isotopologues of LEAADEGSGDMK (from Ogdh) (data shown from day 6), and the resultant labelling curves generated from the acquired data for the peptide across all time-points analysed (MS1 = light grey, SRM = dark grey).

Another advantage of SRM is the selectivity that detection at the product ion level, i.e. MS2, can afford [27]. Many studies have leveraged the selectivity available using high-resolution mass spectrometers to perform MS1 quantification [46–49]. However, co-eluting isobaric interferences can still prevent a clean signal being recorded for a peptide of interest, even using very high resolving powers [50], meaning that inaccuracies and imprecision in measurement can hamper quantification. This is evident in this study when considering the 30 peptides quantified by both MS1 and SRM (electronic supplementary material, figure S7). As an exemplar, interferences contaminated the signal for both the light and heavy isotopologues of the peptide LEAADEGSGDMK (from Ogdh) using MS1 (figure 5, day 6 data shown; electronic supplementary material, figure S8 presents data from six time-points throughout the experiment). This led to inaccurate measurement of the peak areas of the two peptide variants, and thus the RIA at each time-point, resulting in a labelling curve that poorly described the turnover of the parent protein. By using the product ion-level selectivity inherent in the SRM approach, clean signals were obtained for both the light and heavy isotopologues, leading to a more realistically shaped labelling curve, giving confidence that the measurement was correct. The selectivity of a MS measurement is also affected by the variability in chromatographic retention time of different peptides, meaning that (partial) co-elution can occur in one run and not another, leading to further variability in the recording of RIA. This is evident from inspection of the chromatograms of the peptide ELEQIFCQFDSK (from Ogdh) (electronic supplementary material, figure S9, day 6 data shown; electronic supplementary material, figure S10 presents data from six time-points throughout the experiment). The first injection for the MS1 analysis appears to detect a single peak for both the light and heavy isotopologues. Inspection of the data for replicate injections 2 and 3 reveals a significant interfering signal for the light variant of the peptide due to a slight difference in elution times in these analyses. Despite the increased chromatographic separation, the difference was insufficient to achieve baseline resolution, making integration of the peak difficult. As a result, the light peak area was overestimated at each time-point due to a large contribution by the contaminant, leading to an underestimation of RIA (as a result of the inaccurate determination of the denominator of the RIA equation (vide supra)) and thus an erroneous measurement of turnover rate. Using SRM, this interference at the precursor ion level no longer contaminated the peptide signal at the product ion level. Consequently, accurate measurement of peak area for both isotopologues was achieved, producing a labelling curve that was in agreement with other peptides for Ogdh. Overall, the SRM approach produced plots of RIA versus time that gave more confidence in the measurement of protein turnover (electronic supplementary material, figure S7).

The number of peptides measured in this study was greater than might normally be considered necessary to determine the turnover rate of a given protein, a strategy taken to emphasize the ability of SRM to provide consistent quantitative data with greater sensitivity across a number of time-points. For this work, we restricted the analysis to relatively few proteins. To increase multiplexing of a SRM strategy, judicious selection of peptides with good ‘quantotypic’ properties [37] (two or three per protein) would allow high-quality determination of turnover, while expanding the number of proteins measurable in a single experiment. Such a strategy could be applied to turnover measurement of all proteins in a pathway [51] or a large molecular machine [52,53].

## 4. Conclusion

The enhancement of quantitative data quality afforded by targeted SRM analysis compared to non-targeted MS1 quantification in the context of protein turnover measurement has been demonstrated. The dedication of instrument duty cycle to the measurement of specific peptides of interest leads to greater sensitivity, allowing lower abundance peptides and those with poor ionization efficiencies to be detected, and thus turnover data to be gleaned from them. Recording of data at the product ion level, inherent in the SRM approach, can also lead to improved selectivity of measurement, providing more accurate and reliable determination of turnover. This is significant as the improved quantitative data afforded by SRM can be achieved using tandem quadrupole (QqQ) instruments, which are more affordable (approx. £200 000 versus approx. £500 000 for a high mass resolution instrument) and often have a smaller laboratory footprint compared with high mass resolution Orbitrap and quadrupole time-of-flight platforms (many of which are floor standing) used for MS1 quantification and targeted product ion-level analyses related to SRM such as parallel reaction monitoring [54,55], high-resolution multiple-reaction monitoring [56,57] and sequential window acquisition of all theoretical fragment ion spectra [58,59]. Thus, high-quality measurements of protein turnover are available to a large number of proteomics researchers due to the wide availability of QqQ mass spectrometers. This study demonstrates that, while MS1-DDA methods can afford global proteome coverage for protein turnover measurement with minimal experimental design, targeted MS by SRM can enable higher quality data to be obtained for designated cohorts of proteins.

## Supplementary Material

Holman et al., Supplementary Information - Revision 1

## Supplementary Material

Holman et al. - Supplementary Table 1 - Revision 1

## Data accessibility

The MS1-DDA data have been deposited to the ProteomeXchange Consortium via the PRIDE partner repository with the dataset identifier PXD004413 and doi:10.6019/PXD004413. The SRM data have been deposited to PASSEL with the dataset identifier PASS00895 and can be accessed at http://www.peptideatlas.org/PASS/PASS00895.
