# Supplementary material for: Protein turnover measurement using selected reaction monitoring-mass spectrometry (SRM-MS)
Source: Philos Trans A Math Phys Eng Sci. 2016 Oct 28;374(2079):20150362. doi: 10.1098/rsta.2015.0362 (PMC5031629; doi:10.1098/rsta.2015.0362)
Supplement: Holman et al., Supplementary Information - Revision 1 [file rsta20150362supp1.docx]

Protein turnover measurement using selected reaction monitoring-mass spectrometry (SRM-MS)

Stephen W. Holman^1^**^‡^***, Dean E. Hammond^2^**^‡^**, Deborah M. Simpson^1^, John Waters^3^, Jane L. Hurst^3^ and Robert J. Beynon^1^*

^1^Centre for Proteome Research, Department of Biochemistry, Institute of Integrative Biology, University of Liverpool, Crown Street, Liverpool, L69 7ZB, UK

^2^Cellular and Molecular Physiology, Institute of Translational Medicine, University of Liverpool, Crown Street, Liverpool, L69 3BX, UK

^3^Mammalian Behaviour and Evolution Group, Department of Evolution, Ecology and Behaviour, Institute of Integrative Biology, University of Liverpool, Leahurst Campus, Neston, CH64 7TE, UK

^‡^ These authors contributed equally to the manuscript.

*Corresponding authors: Professor Robert J. Beynon

Email: [r.beynon@liverpool.ac.uk](mailto:r.beynon@liverpool.ac.uk)

Tel: +44 151 794 4312

Dr. Stephen W. Holman

Email: [stephen.holman@liverpool.ac.uk](mailto:stephen.holman@liverpool.ac.uk)

Tel: +44 151 794 5344

**Supplementary Figure Legends**

**
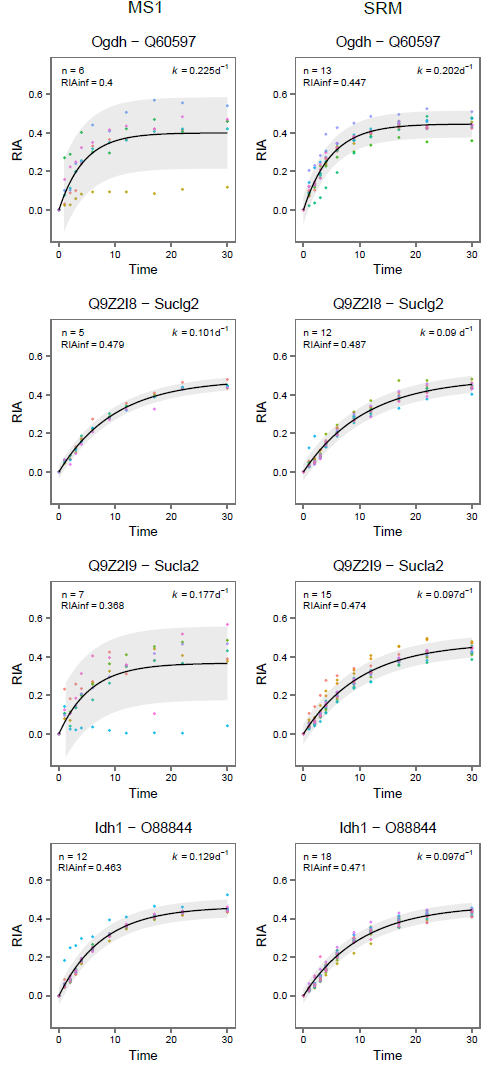
**

**Figure S1 | Labelling trajectories and non-linear curve fitting for proteins using MS1 and SRM**

For four proteins, the labelling trajectory for multiple peptides (number of peptides is given by n) was calculated for all peptides. Each datum is the average of RIA values determined for each peptide from triplicate analyses at a given time-point, plotted against time in days. For the entire data set, non-linear curve fitting was applied to recover the best fit values for *k*_deg_ and the plateau value of RIA (RIA_inf_). The line of best fit is included and the grey region defines the 95% confidence limits on the fitted curve.


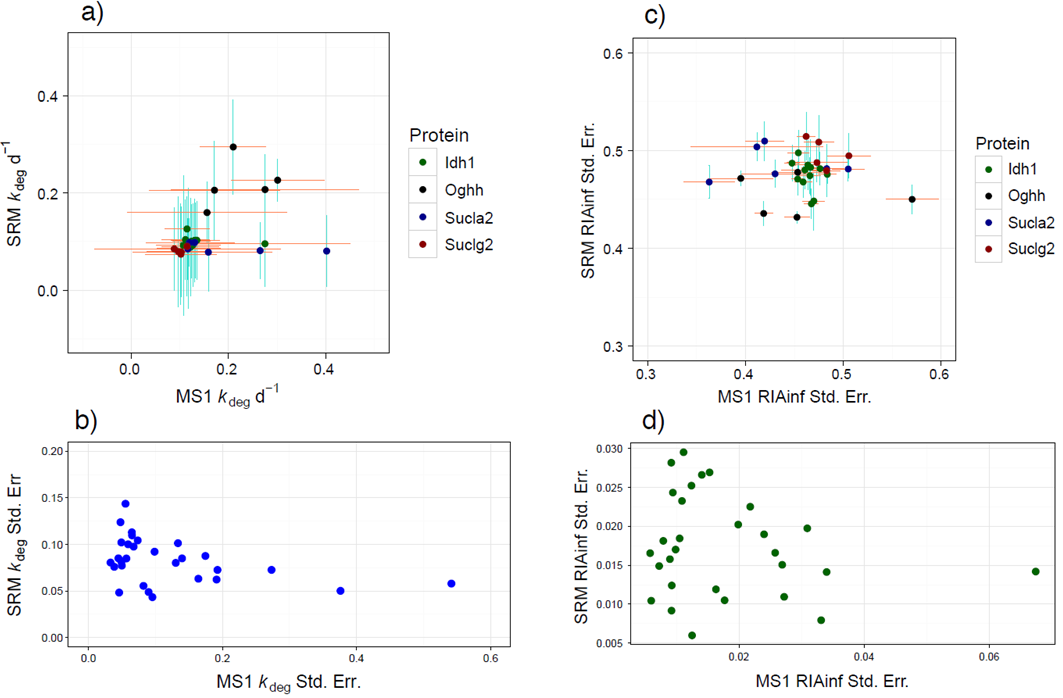


**Figure S2 | Error performance of MS1 and SRM methods**

For four proteins, non-linear curve fitting was used to recover the values of *k*_deg_ and the value of the RIA value at plateau (RIA_inf_) from peptides that were quantified by both MS1 and SRM. Panels a) and c): scatterplot of *k*_deg_ and RIA_inf_ values ± standard error of the mean for the peptides quantified by both MS1 and SRM. Data points are the best fit solution for parameter estimates with solid lines delineating ± standard error of the non-linear curve fit. Panels b) and d): Scatterplot of the errors in each parameter.

**Figure S3 | Peptide labelling curves generated using MS1**

Labelling trajectories (RIA, t) for all peptides quantified by MS1. Each datum is the average of RIA values determined for each peptide from triplicate analyses at a given time-point, plotted against time in days. For each peptide, non-linear curve fitting was applied to recover the best fit values for *k*_deg_ and the plateau value (denoted Asym) of RIA (RIA_inf_). The line of best fit is included.

**Figure S4 | Peptide labelling curves generated using SRM**

Labelling trajectories (RIA, t) for all peptides quantified by SRM. Each datum is the average of RIA values determined for each peptide from triplicate analyses at each time-point, plotted against time in days. For each peptide, non-linear curve fitting was applied to recover the best fit values for *k*_deg_ and the plateau value (denoted Asym) of RIA (RIA_inf_). The line of best fit is included.

**
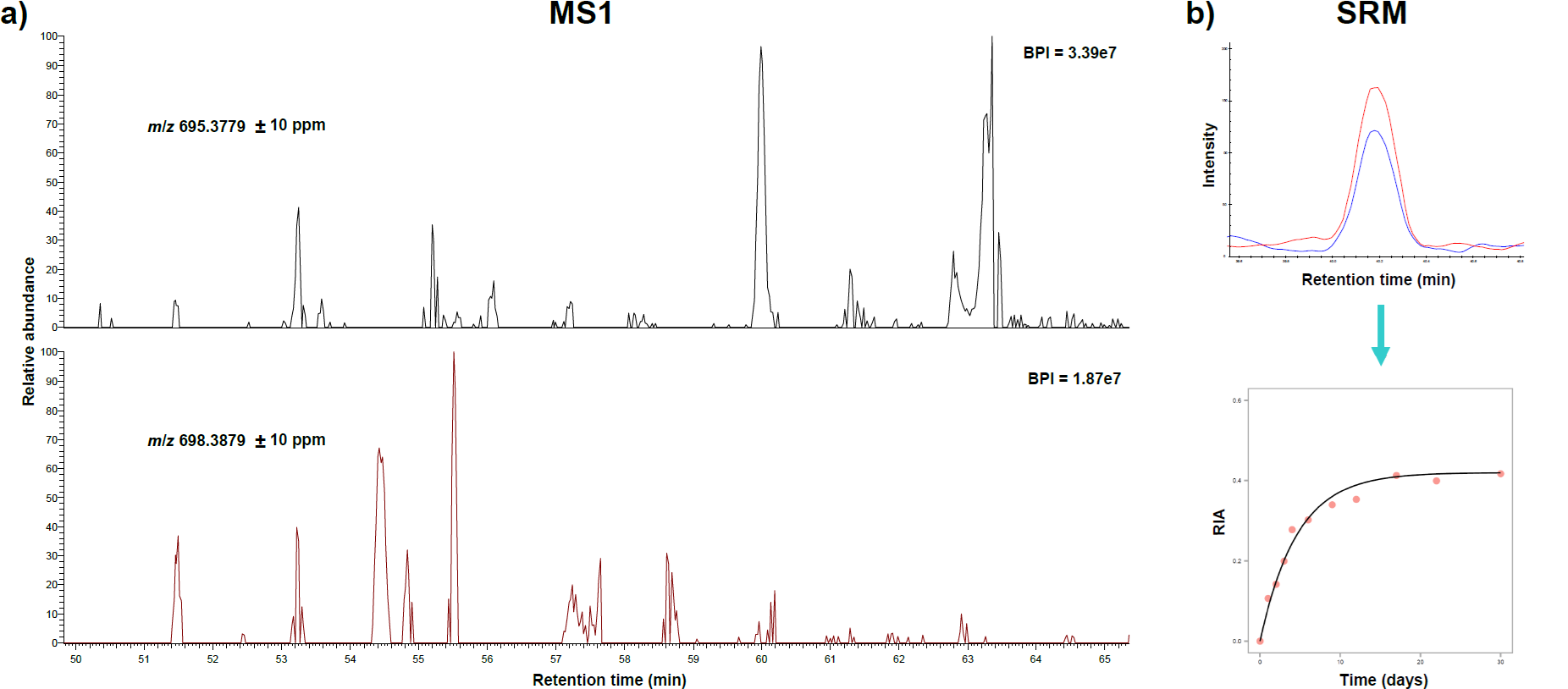
**

**Figure S5** **| Performance of a selected peptide in MS1 compared to SRM**

Panel a) MS1 reconstructed ion current chromatograms of light (*m*/*z* 695.3779) and heavy (*m*/*z* 698.3879) AAAQVLGNSGLFNK (from Sucla2) with a precursor ion *m*/*z* tolerance of ± 10 ppm showing no evidence of either isotopologue. Panel b): SRM chromatogram of light (red) and heavy (blue) AAAQVLGNSGLFNK (from Sucla2), showing co-eluting peaks for both, and the resulting labelling curve plotted from the acquired data for the peptide across all time-points analysed. All data are from injection 1 of the day 22 sample. BPI = Base peak ion intensity.

**
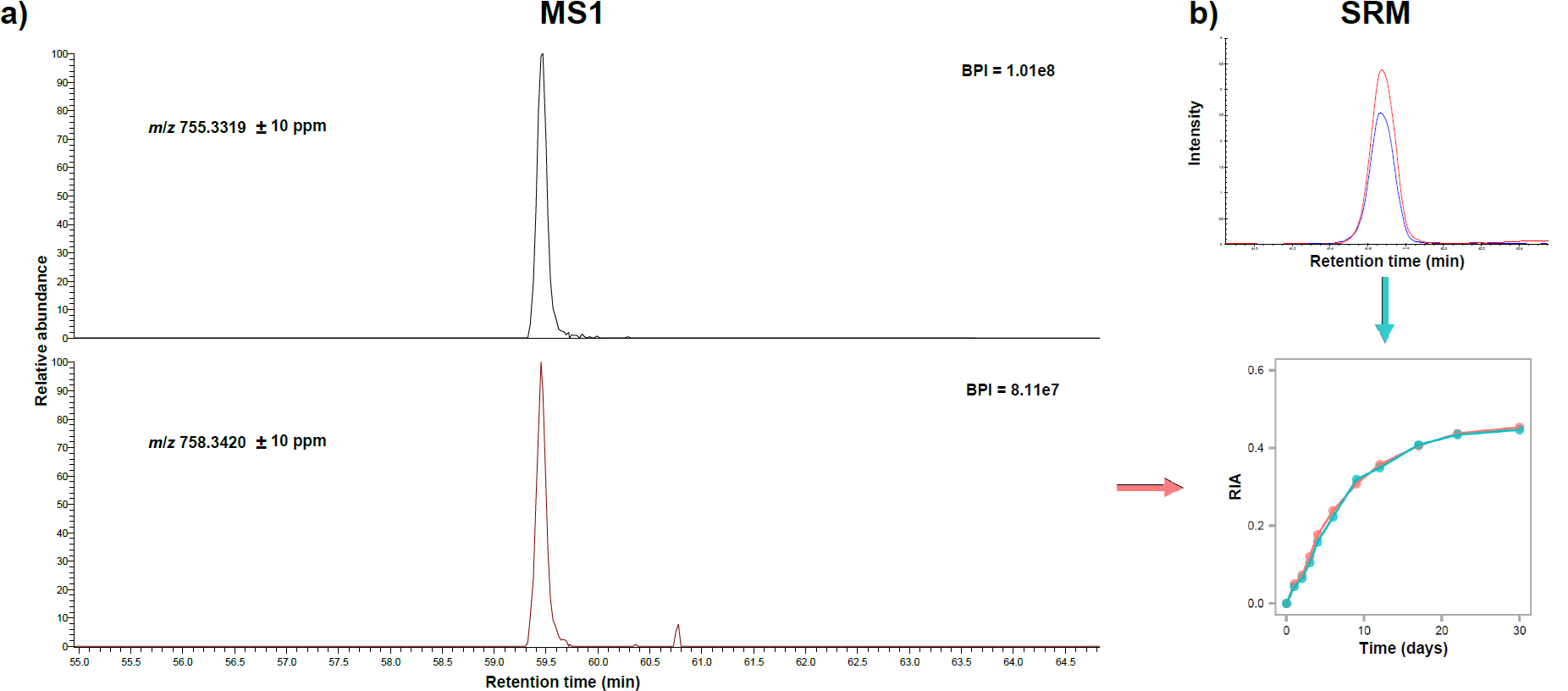
**

**Figure S6 | Performance of SDYLNTFEFMDK using MS1 and SRM**

a) MS1 reconstructed ion current chromatograms of light (*m*/*z* 755.3319) and heavy (*m*/*z* 758.3420) SDYLNTFEFMDK (from Idh1) with a precursor ion *m*/*z* tolerance of ± 10 ppm, and b) SRM chromatogram of light (red) and heavy (blue) SDYLNTFEFMDK (from Idh1), both showing co-eluting peaks for each isotopologue, and the resulting plots of RIA against time (days) from the acquired data for the peptide across all time-points analysed. Both data are from injection 1 of the day 22 sample. BPI = Base peak ion intensity.

**Figure S7 | Plots of (RIA, t) for all peptides quantified using both MS1 and SRM**

RIA plotted against time (days^-1^) for all peptides quantified in both MS1 and SRM experiments ((MS1 = coral, SRM = turquoise).

**
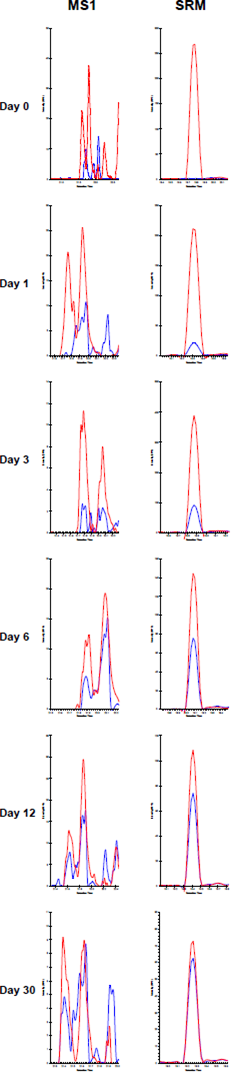
**

**Figure S8 | MS1 and SRM chromatograms of LEAADEGSGDMK**

MS1 and SRM chromatograms for the light (red) and heavy (blue) isotopologues of LEAADEGSGDMK (from Ogdh) over the course of the experiment (days 2, 4, 9 17 and 22 removed for clarity). Each chromatogram is from the first of the triplicate analyses.


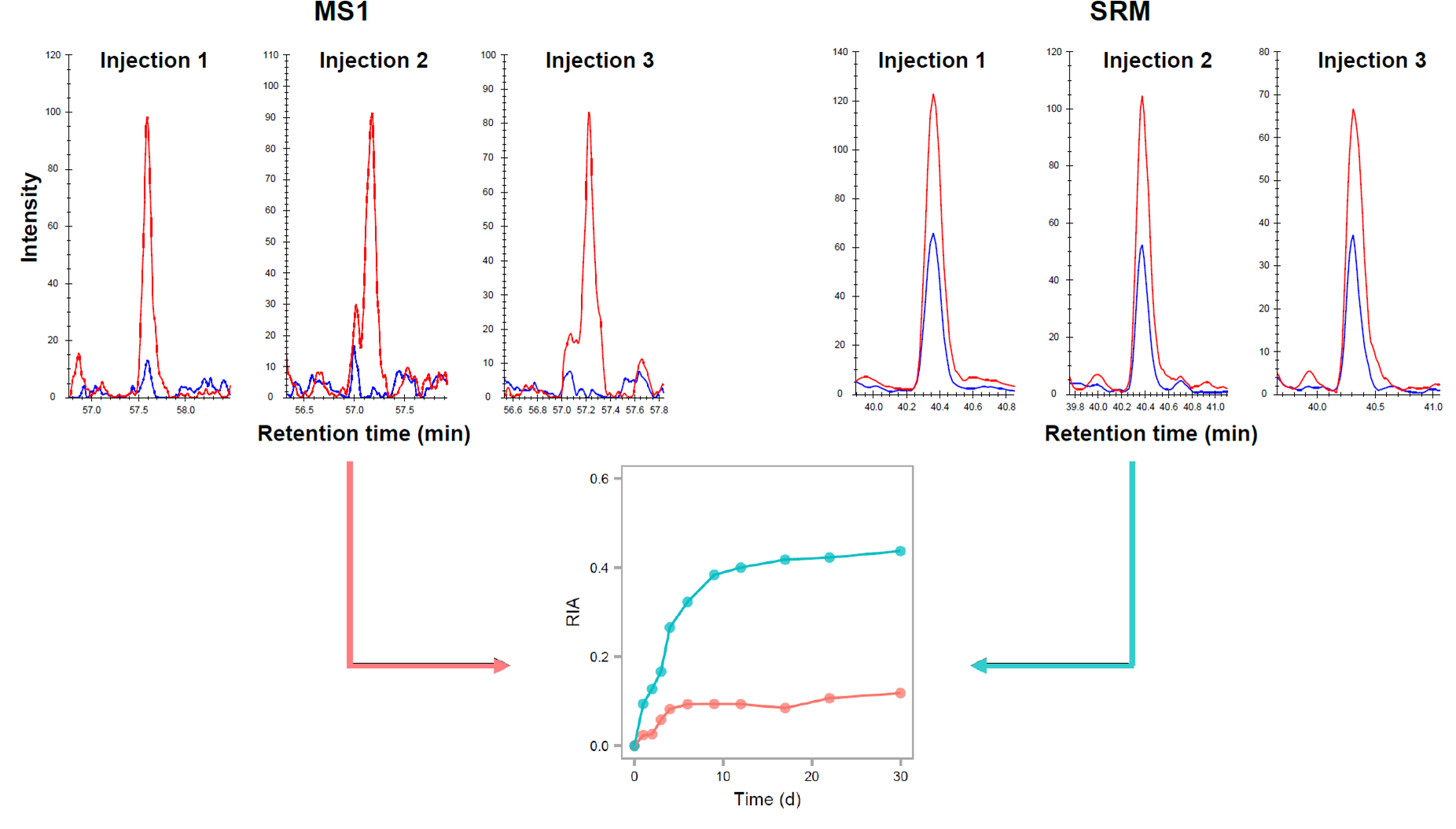


**Figure S9 | Contamination of peptide chromatograms in MS1 compared to SRM**

Triplicate MS1 reconstructed ion current chromatograms and triplicate SRM chromatograms for the light (red) and heavy (blue) isotopologues of ELEQIFCQFDSK (from Ogdh) (data shown from day 6), and the resultant labelling curves generated from the acquired data for the peptide across all time-points analysed (MS1 = coral, SRM = turquoise).


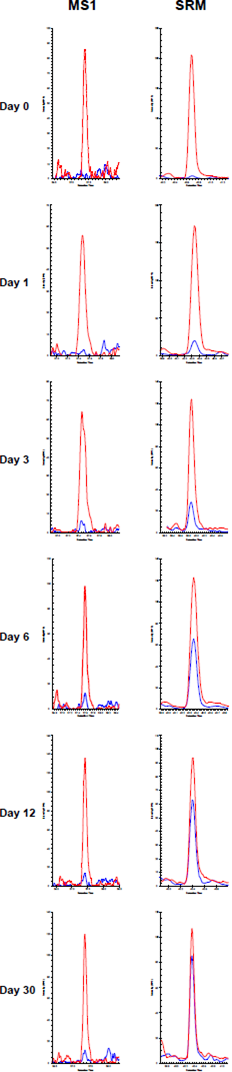


**Figure S10 | MS1 and SRM chromatograms of ELEQIFCQFDSK**

MS1 and SRM chromatograms for the light (red) and heavy (blue) isotopologues of ELEQIFCQFDSK (from Ogdh) over the course of the experiment (days 2, 4, 9 17 and 22 removed for clarity). Each chromatogram is from the first of the triplicate analyses.
